# Extremely Sensitive Microwave Microfluidic Dielectric Sensor Using a Transmission Line Loaded with Shunt LC Resonators

**DOI:** 10.3390/s21206811

**Published:** 2021-10-13

**Authors:** Haneen Abdelwahab, Amir Ebrahimi, Francisco J. Tovar-Lopez, Grzegorz Beziuk, Kamran Ghorbani

**Affiliations:** School of Engineering, RMIT University, Melbourne, VIC 3001, Australia; s3753004@student.rmit.edu.au (H.A.); francisco.tovarlopez@rmit.edu.au (F.J.T.-L.); greg.beziuk@rmit.edu.au (G.B.); kamran.ghorbani@rmit.edu.au (K.G.)

**Keywords:** dielectric measurement, microfluidic sensor, microwave sensor, planar resonators

## Abstract

In this paper, a very high sensitivity microwave-based planar microfluidic sensor is presented. Sensitivity enhancement is achieved and described theoretically and experimentally by eliminating any extra parasitic capacitance not contributing to the sensing mechanism. The sensor consists of a microstrip transmission line loaded with a series connected shunt LC resonator. A microfluidic channel is attached to the area of the highest electric field concentration. The electric field distribution and, therefore, the resonance characteristics are modified by applying microfluidic dielectric samples to the sensing area. The sensor performance and working principle are described through a circuit model analysis. A device prototype is fabricated, and experimental measurements using water/ethanol and water/methanol solutions are presented for validation of the sensing mathematical model.

## 1. Introduction

Today, microwave sensors are attracting increased interest among the industry and researchers due to their low-cost, compact size, compatibility with the wireless electronics, and the developing fabrication technologies, such as additive manufacturing [1,2,3,4,5,6,7]. As a main application, microwave sensors have been designed and applied in materials characterization because of their high sensitivity to the electromagnetic properties of various materials [8,9,10,11,12,13,14,15,16,17,18,19,20,21,22,23,24,25,26,27,28,29,30,31,32].

Both resonant and non-resonant devices were designed for materials characterization at microwave frequencies. The non-resonant sensors are mainly designed using transmission lines and waveguides, where loading the material-under-test (MUT) modifies the guided wave impedance and/or the wave propagation constant. The phase and amplitude variations, in turn, alter the transmission/reflection amplitude and phase response of the sensor over a wide frequency range. These variations can be translated to the complex dielectric or magnetic properties of the MUTs [33,34]. On the other hand, by loading MUTs on the resonant microwave sensors, the resonance frequency, quality factor, or transmission/reflection amplitude at the resonance frequency is modified. These modifications can be measured and translated to the electromagnetic properties of the MUTs around the resonance frequency of the sensor. Generally, resonance-based microwave sensors offer a more accurate measurement over a narrower frequency range in comparison with wideband sensors. This is essentially because of the high quality factor resonance and highly dense electromagnetic fields produced by the resonant sensors, which is highly sensitive to any dielectric/magnetic material loading [35,36].

Microfluidic dielectric sensors are one of the most prevalent microwave sensors. These sensors offer complex dielectric measurements of fluidic samples at microwave frequencies, where a very little amount of sample is required for sensing by virtue of microfluidic technology. By fabricating tiny channels, liquid samples can be guided to the sensing area and processed in micro and nano scales. Microfluidic dielectric sensors have found applications in the characterization and identification of organic chemicals and biological samples [37,38,39,40,41]. A very high sensitivity to dielectric loadings can be achieved using three-dimensional (3D) cavity resonators [42]. However, such structures are bulky compared to their planar counterparts. On the other hand, planar sensors offer more capability with the integrated platforms. More specifically, microwave planar microfluidic sensors are of great interest because of their high compatibility of integration in lab-on-a-chip technology [43,44,45,46]. However, the main challenge in planar structures is a low quality factor resonance and a smaller fringing electric field (E-field) compared to the non-planar waveguide approaches that results in a lower sensitivity in planar sensors. In order to address this challenge, complementary split-ring resonators (CSRRs) were proposed in Reference [47] to increase the fringing E-field and to offer higher sensitivity. In Reference [48], a LC resonator embedded in a coplanar waveguide (CPW) was used to increase the fringing E-field and higher sensitivity. An interdigital capacitor as a capacitive gap in a split-ring resonator (SRR) is used to increase the fringing field and achieve a larger effective sensing area in Reference [49]. A similar approach was used in References [50,51] by using meandered slots in the CSRR and complementary electric-LC (CELC) resonators to achieve higher E-field concentration and better sensitivity. In Reference [52], a negative refractive index transmission line is used as a coupling structure to improve sensitivity in a SRR-based microfluidic sensor.

However, in all of these sensors, there are parasitic capacitances that contribute to the resonance frequency, but they do not contribute to sensing. This means, applying the MUT to the sensing area does not affect these parasitic capacitances. This is the main limiting factor in sensitivity degradation. Our recently published work in Reference [53] studied this effect, where a very high sensitivity microwave microfluidic dielectric sensor was designed using a microstrip transmission line loaded with a step impedance resonator (SIR). The effect of parasitic capacitances was suppressed for sensitivity improvement by etching a slot out of the ground plane below the SIR. However, there was still small parasitic capacitance since the microfluidic channel was not covering the whole capacitive gap area. Furthermore, the sensor sensitivity was adversely affected by a small fringing parasitic capacitance between the inductive trace and the ground plane. Here, we propose a new microwave microfluidic sensor using a parallel LC resonator, connected in series with the feed microstrip transmission line. The capacitance is implemented with two triangular patches placed with a small capacitive gap in between. The inductance is realised using a thin metallic trace connected in parallel to the capacitive patches. All the parasitic capacitances are removed by etching a window out of the ground plane below the resonator. A microfluidic channel is designed and aligned with the capacitive gap between the patches. The microfluidic liquid sample in the channel is in contact with the capacitive patches of the sensor. In this way, the whole capacitive element in the sensor is modified by applying liquids into the channel resulting in an extremely sensitive device. The proposed sensor offers almost more than twofold higher sensitivity than the one in Reference [53]. To the best of our knowledge, it offers the highest sensitivity compared to the state-of-the-art planar frequency shift microfluidic dielectric sensors in the literature. The design procedure and the equivalent circuit model analysis of the proposed sensor will be presented in Section 2. Section 3 presents verification of the proposed sensing concept through the measurement results of a fabricated sensor prototype and a comparison with the state-of-the-art designs in the literature. Finally, Section 4 presents the main conclusions.

## 2. Sensor Design and Operation Principle

### 2.1. Design Principle and Circuit Model

**Figure 1:**
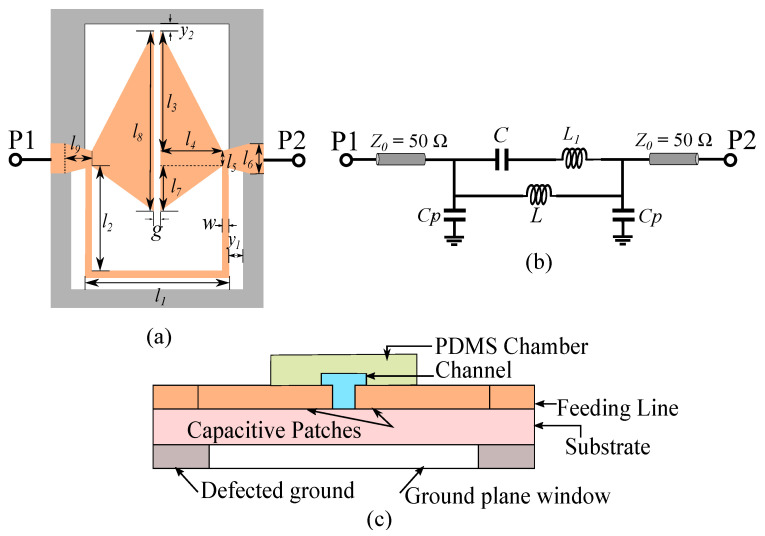
(**a**) Layout of the sensor developed for high-sensitivity dielectric sensing. (**b**) Equivalent circuit model of the proposed sensor. (**c**) Cross sectional view of the proposed sensor. The geometrical dimensions are: g = 0.2 mm, w = 0.2 mm, $l_{1}$ = 5.4 mm, $l_{2}$ = 3.8 mm, $l_{3}$ = 4.4 mm, $l_{4}$ = 2.4 mm, $l_{5}$ = 0.6 mm, $l_{6}$ = 1 mm, $l_{7}$ = 1.6 mm, $l_{8}$ = 6.6 mm, $l_{9}$ = 1 mm, $y_{1}$ = 0.6 mm, $y_{2}$ = 0.4 mm.

A two-dimensional (2D) layout of the proposed sensor is shown in Figure 1, together with its cross-sectional view and equivalent circuit model. The sensor consists of a parallel LC resonator loaded in series to the host microstrip transmission line. An equivalent circuit model of the designed sensor is presented in Figure 1b. A window has been opened in the ground plane below the resonator. This helps in boosting up the value of the U-shaped inductance made of the thin metallic trace and removes the parasitic capacitances with the ground plane. This way, the parasitics not contributing to sensing are removed to enhance the sensitivity to dielectric loadings into the capacitive gap. Such a strategy was used in Reference [53], where there was still additional parasitic capacitance between the capacitive patch and ground plane implemented on the top of the substrate that degrade the sensitivity. The current design removes all the possible parasitic capacitances by etching a window in the ground plane, as shown in Figure 1a.

In the circuit model, the *L* inductance represents the inductive behavior of the U-shaped thin metallic trace of width *w*. The *C* capacitor models the series gap capacitance between the two triangular metallic patches, and $L_{1}$ is the parasitic series inductance of the triangular patches. The host miscrostrip line is represented with short 50Ω transmission line sections on sides, and the $C_{p}$ capacitances model the shunt capacitances between the ground plane and feed lines at the feed connection points. The value of these two capacitors is very small because of the defected ground in the bottom layer.

The four unknown of the circuit model (*L*, *C*, $L_{1}$, and $C_{p}$) can be calculated based on the following four criteria:

(i) The stopband in the transmission response ($S_{21}$) happens, when the combination of *L*, $L_{1}$, and *C* is open circuit. This happens at

(1) $$f_{z}=\frac{1}{2\pi }\sqrt{\frac{1}{C\left(L+L_{1}\right)}}.$$

(ii) The $S_{11}$ curve intersects with the unit admittance circle in the Smith Chart at the resonance frequency of the $L_{1}C$ tank described by:

(2) $$f_{p}=\frac{1}{2\pi }\sqrt{\frac{1}{CL_{1}}}.$$

(iii) The input admittance seen by each of the ports at $f_{p}$ equals to:

(3) $$Y_{in}\left(f_{p}\right)=j4\pi f_{p}C_{p}+\frac{1}{Z_{0}}.$$

(iv) At the frequency, where the phase of the transmission coefficient reaches 90${}^{\circ }$ ($\phi_{90^{\circ }}$), the shunt and series impedances are complex conjugate. This means:

(4) $$Z_{s}\left(f_{90^{\circ }}\right)=-jZ_{p}\left(f_{90^{\circ }}\right),$$

where $Z_{s}$ is the equivalent impedance by combination of *C*, *L*, and $L_{1}$, and $Z_{p}$ is the impedance of the parallel capacitance $C_{p}$.

**Figure 2:**
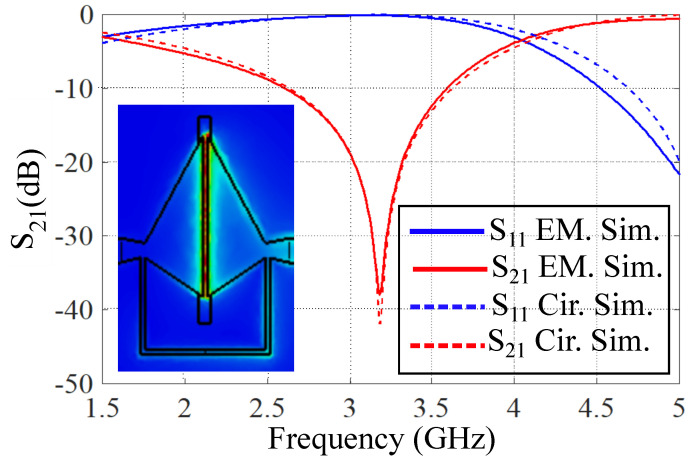
Comparison between the full–wave electromagnetic and the equivalent circuit model simulation results of the designed sensor. The extracted values of lumped circuit elements are: *L* = 7.79 nH, *C* = 0.195 pF, $L_{1}$ = 5.03 nH, $C_{p}$ = 0.03 pF, and θ = 8${}^{\circ }$.

Solving Equations (1)–(4) simultaneously yields the values of the four lumped elements (*C*, *L*, $L_{1}$, $Z_{p}$, and $C_{p}$) in the circuit model in Figure 1b. A comparison between the S-parameters of the bare sensor obtained from the full-wave electromagnetic simulations and the ones obtained from the circuit model simulations is presented in Figure 2. A good agreement between the circuit model and full-wave simulations results verifies the developed circuit model.

### 2.2. Sensitivity Analysis

Sensitivity of the design sensor is defined as:

(5) $$S=\frac{df_{z}}{dC}\frac{dC}{d\varepsilon_{LUT}},$$

where $\varepsilon_{LUT}$ is the relative permittivity of the liquid-under-test (LUT) in the microfluidic channel. Using (1), the first term of the sensitivity is calculated as:

(6) $$\frac{df_{z}}{dC}=\frac{-1}{4\pi \sqrt{\left(L+L_{1}\right)C^{3}}}.$$

Furthermore, assuming a semi-infinite dielectric substrate [54], by loading a dielectric liquid into the channel, the capacitance will be modified to:

(7) $$C_{LUT}=C_{empty}\frac{\varepsilon_{r,eq}+\varepsilon_{LUT}}{\varepsilon_{r,eq}+1},$$

where $C_{empty}$ is the capacitance of the unloaded sensor, and $\varepsilon_{r,eq}$ is the equivalent relative permittivity of the semi-infinite sensor substrate by considering the effect of microfluidic channel [54]. Thus, the second term of the sensitivity is obtained as:

(8) $$\frac{dC}{d\varepsilon_{LUT}}=\frac{C_{empty}}{\varepsilon_{r,eq}+1}.$$

Using (6) and (8), the sensitivity (*S*) is calculated as:

(9) $$S=\frac{-1}{4\pi \sqrt{\left(L+L_{1}\right)C_{empty}\frac{{\left(\varepsilon_{r,eq}+\varepsilon_{LUT}\right)}^{3}}{\varepsilon_{r,eq}+1}}}.$$

Based on the calculated sensitivity, considering a predetermined resonance frequency of the unloaded sensor, a smaller capacitance of the unloaded sensor results in better sensitivity. This means that a larger capacitive gap will offer better sensitivity. However, a larger capacitive gap (smaller $C_{empty}$) causes a narrow bandwidth resonance that will be easily damped by applying lossy liquid samples, such as ethanol, to the channel, causing a false detection. Therefore, a small gap (g=0.2 mm) is considered in the design to ensure a detectable resonance, even for highly lossy samples.

**Figure 3:**
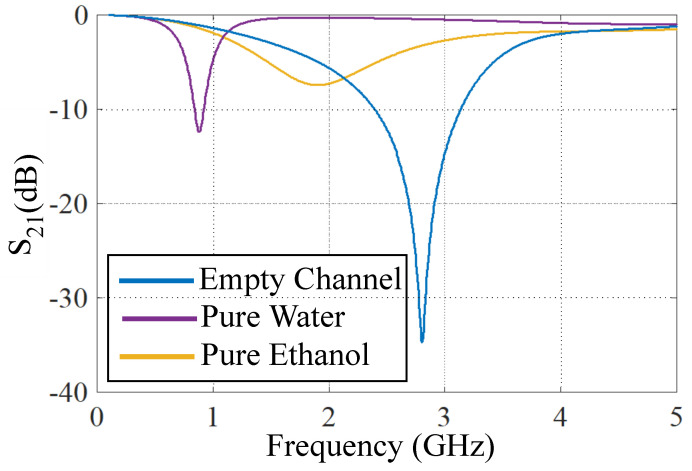
Transmission response of the sensor for: an empty channel, channel filled with water, and a channel filled with pure ethanol.

The designed sensor can be used for the dielectric characterization of microfluidic samples by implementing a microfluidic channel in the area with the highest electric field concentration. Based on the simulation results of a bare sensor in Figure 2, there is a highly dense E-field concentration in the capacitive gap area between the two rectangular patches. Thus, a microfluidic channel is implemented along the gap area. By applying microfluidic dielectric samples to the channel, both the notch depth and center frequency of resonance will be modified from which the dielectric properties of the sample can be obtained. This is shown in Figure 3, where the simulated responses of an empty channel, a channel filled with water, and a channel filled with pure ethanol.

According to (1), the resonance frequency will change with the change of *C*, $L_{1}$, and $L_{2}$ elements. In the designed sensor, changing the liquid sample modifies the overall permittivity of the medium within the microfluidic channel, which, in turn, alters the value of the gap capacitance between the triangular metallic patches *C*. All the parasitic capacitances are removed by considering a window in the ground plane below the designed resonator, and *C* is the only capacitance contributing to sensing. This guarantees a very high sensitivity based on the explanations in the Section 1.

## 3. Fabrication and Validation of the Microfluidic Sensor

### 3.1. Fabrication of the Microfluidic Sensor

**Figure 4:**
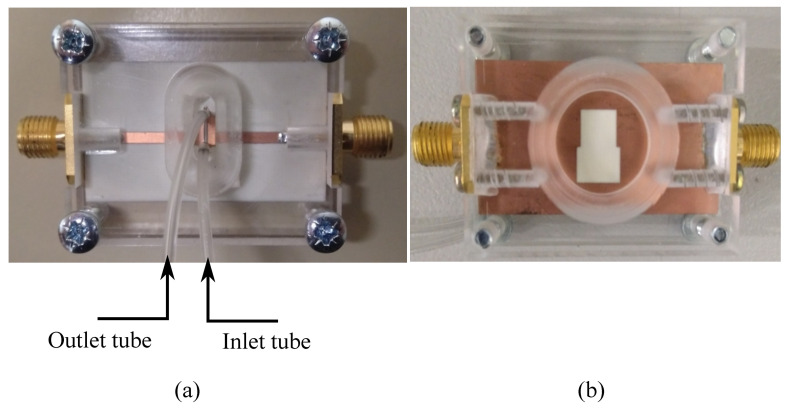
The fabricated microwave microfluidic sensor. (**a**) Front view. (**b**) Back view.

A sensor prototype is fabricated on a 0.762 mm thick Rogers RO4350B with $\varepsilon_{r}\;=\;3.66$, tanδ=0.0037. The microfluidic channel is fabricated using established soft photolithography methods. Briefly, using maskless lithography (MLA 150, Heidelberg instruments, Germany), a master template was fabricated onto a 4-inch silicon wafer using a (SU-8 3050) photoresist (MicroChem Corp, MA, USA ) to produce defined channel features with a height of 70 µm. Polydimethylsiloxane (PDMS), which is a biocompatible, durable, and easy to process polymer for microfluidic devices (Sylgard 184), was mixed with a curing agent in a 1:10 ratio by weight and cast on the master template and cured at 80 ${}^{\circ }$C in a convection oven for 20 min. The cured PDMS was peeled off the mold and punched with a 0.75 mm hole at the inlet and outlets to connect in/out microfluidic tubes. The microfluidic channel’s width, length, and height are 0.65, 6.5, and 0.07 mm, respectively. A photograph of the fabricated and assembled sensor is presented in Figure 4.

**Figure 5:**
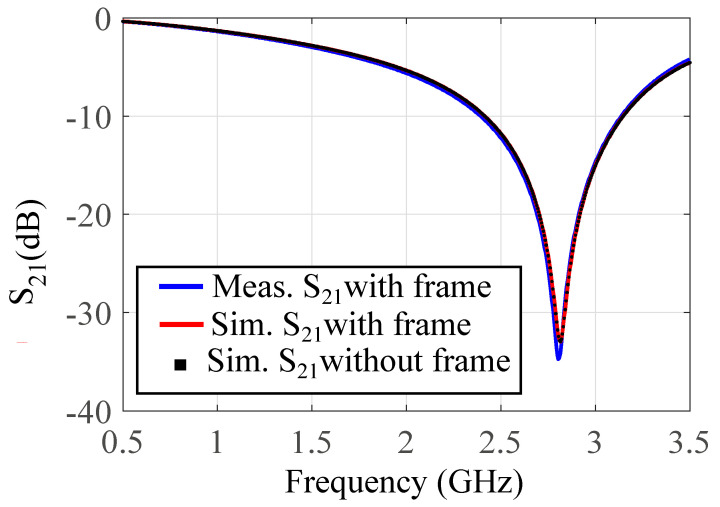
Measured and simulated transmission response of the sensor (an empty channel) with and without the plexiglass frame.

The PDMS channel is manually aligned to the sensing area, and a syringe, together with microfluidic tubes, is attached to the in/outlet of the channel for delivering the samples to the sensing area. A plexiglass frame was milled to fit the sensor shape. It was used, together with four screws, to push the PDMS channel against the sensor to preserve it from deformation and miss-alignment during the measurements. A hole is milled out of the frame beneath the ground plane window to ensure that the frame dielectric properties does not affect the sensor response. In order to make sure that the plexiglass frame does not affect the sensor response, a comparison is performed between the simulated results of the bare sensor, the sensor with the plexiglass frame, and the measured transmission response of the final fabricated sensor inside the frame. Based on Figure 5, the transmission responses of the three cases are almost identical, confirming that the plexiglass frame does not affect the sensor performance.

### 3.2. Measurement and Verification

**Figure 6:**
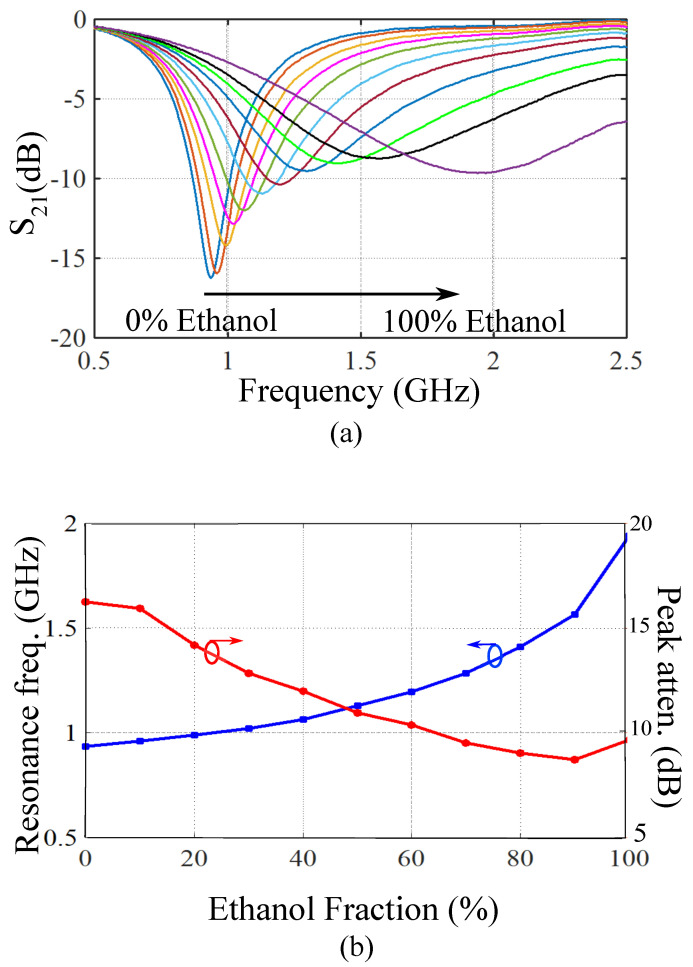
(**a**) Measured transmission responses of the sensor for water/ethanol samples with various ethanol volume fractions. The variation of ethanol volume fraction in each step is 10%. (**b**) The resonance frequency and the peak attenuation for different ethanol volume fractions.

Experimental measurements of the fabricated sensor prototype are presented in this section to verify the proposed microfluidic dielectric sensing concept. In order to characterize the dielectric properties of aqueous solutions, the designed sensor is first calibrated using water/ethanol solutions of various ethanol volume fractions to study the change of complex permittivity $(\varepsilon_{r}^{\prime }$−$j\varepsilon_{r}^{\prime \prime })$ with various channel loading. Water/ethanol solutions are chosen as they offer a wide range of relative permittivity values ideal for calibration. The measured transmission responses of the sensor for water/ethanol samples are plotted against frequency in Figure 6a. Measurements are performed by filling the channel with the sample liquid and measuring the corresponding transmission response $\left(S_{21}\right)$ using a vector network analyzer (VNA). Then, the channel is drained for the next channel loading. From the plots, both the resonance frequency and the peak attenuation ($|S_{21\min}|$) are affected by loading different dielectric fluids to the channel. The variations of the resonance frequency and the peak attenuation as a function of the ethanol volume fraction are presented in Figure 6b.

A mathematical relation is derived using the non-linear least square method in MATLAB to relate the variation of the resonance frequency and the peak attenuation to the complex permittivities of the water/ethanol solutions. The obtained mathematical relation is:

(10) $$\left[\begin{array}{c} \Delta f_{0}\\ \Delta |S_{21}| \end{array}\right]=\left[\begin{array}{cc} -0.0163 & -0.035\\ 0.09482 & -0.4311 \end{array}\right]\left[\begin{array}{c} \Delta \varepsilon_{sam}^{\prime }\\ \Delta \varepsilon_{sam}^{\prime \prime } \end{array}\right],$$

where $\Delta f_{0}$ = $f_{sam}-f_{ref}$, $\Delta |S_{21}|$ = $|S_{21sam}\left|-\right|S_{21ref}|$. The subscript “sam” refers to the sample under test, and “ref” to the reference mixture. The reference mixture is the pure ethanol. $\Delta f_{0}$ and $\Delta |S_{21}|$ are obtained from the measurement data set shown in Figure 6. $\Delta \varepsilon_{sam}^{\prime }$= $\varepsilon_{sam}^{\prime }$−$\varepsilon_{ref}^{\prime }$, and $\Delta \varepsilon_{sam}^{\prime \prime }$ = $\varepsilon_{sam}^{\prime \prime }$−$\varepsilon_{ref}^{\prime \prime }$. Based on (10), a model is derived that relates the complex permittivity to resonance frequency and peak attenuation as follows:

(11) $$\left[\begin{array}{c} \Delta \varepsilon_{sam}^{\prime }\\ \Delta \varepsilon_{sam}^{\prime \prime } \end{array}\right]=\left[\begin{array}{cc} -36.5934 & 4.9658\\ -8.0488 & 1.2274 \end{array}\right]\left[\begin{array}{c} \Delta f_{0}\\ \Delta |S_{21}| \end{array}\right].$$

Using (11), the complex dielectric properties of any unknown fluid sample can be derived by measuring its corresponding resonance frequency and peak attenuation.

### 3.3. Validation of the Sensing Model

**Figure 7:**
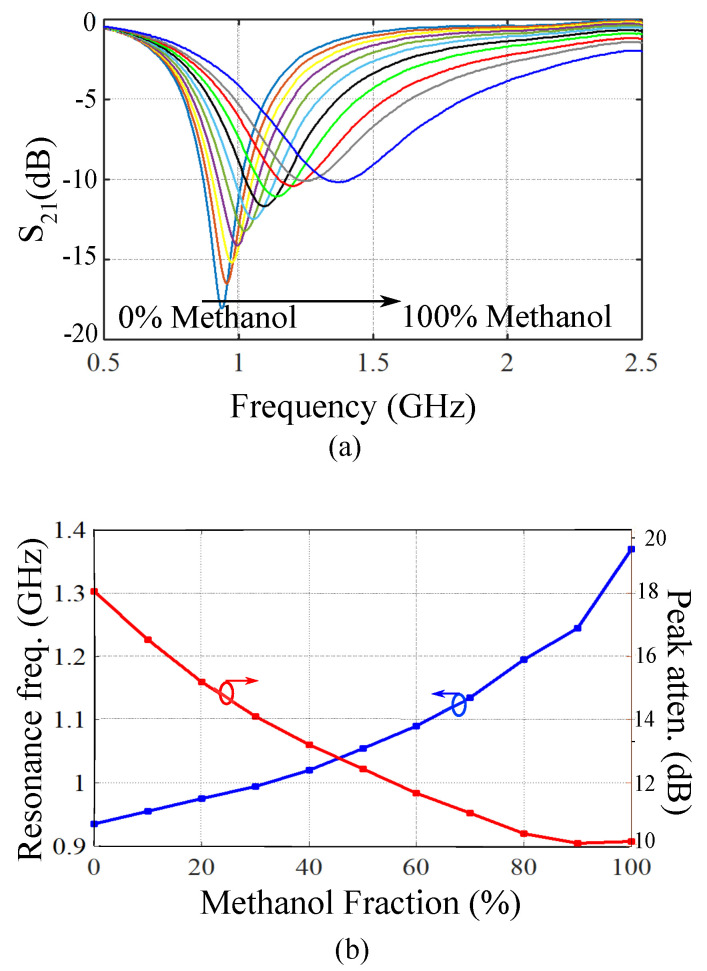
(**a**) Measured transmission responses of the sensor for water/methanol samples with various methanol volume fractions. The variation of methanol volume fraction in each step is 10%. (**b**) The resonance frequency and the peak attenuation for different methanol volume fractions.

**Figure 8:**
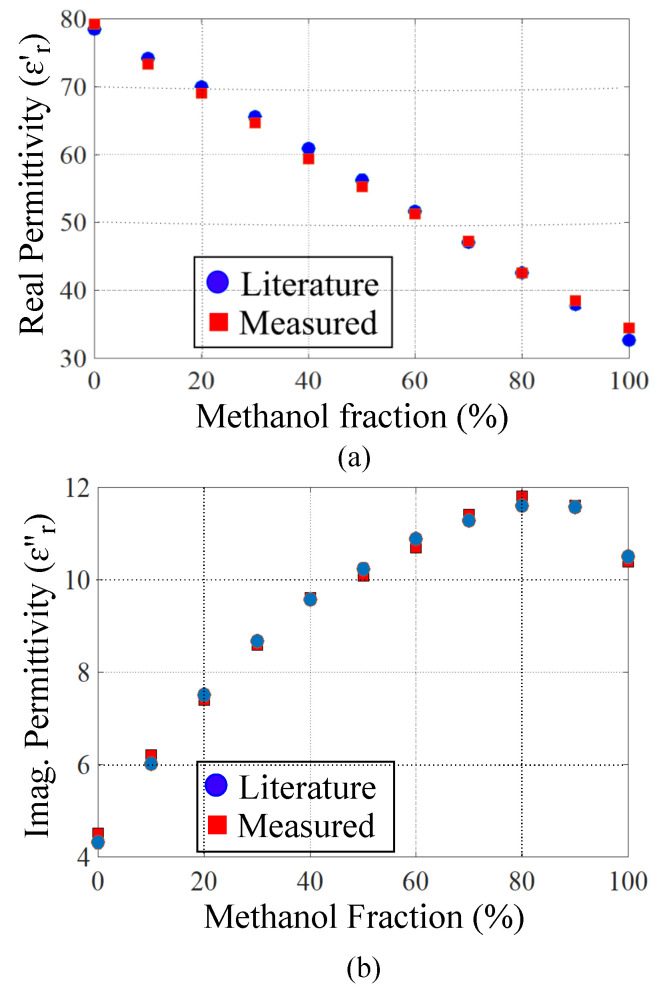
Comparison between the actual values of the relative permittivity from Reference [55] and the ones measured using the designed sensor. (**a**) The real part of the relative permittivity. (**b**) The imaginary part of the relative permittivity.

The developed complex permittivity measurement model in (11) is verified in this section by calculating the complex permittivity values of water/methanol mixture based on measuring resonance frequency and peak attenuation of each sample. The measured transmission responses of the sensor for water/methanol solutions are presented in Figure 7a, whereas the measured resonance frequencies and peak attenuations for each water/methanol variation are plotted in Figure 7b as a function of methanol volume fraction. The sensing model in (11) is now used to calculate the complex dielectric properties of water/methanol samples based on the measured resonance frequency and peak attenuations. A comparison between the measured values of the relative permittivities and the actual ones mentioned in Reference [55] is presented in Figure 8. Good agreement between the measured relative permittivities and the actual values in Reference [55] validates the accuracy of the developed sensing model in (11) and the proposed sensor design.

### 3.4. Comparison with Other Planar Sensors

This section presents comparisons between the presented sensor with the state-of-the-art resonance-based planar microwave microfluidic sensors. The comparison criteria are mainly based on sensor sensitivity. The sensitivity in resonance-based microfluidic dielectric sensors is defined as

(12) $$S=|\frac{f_{\varepsilon_{r}}-f_{empty}}{f_{empty}\left(\varepsilon_{r}-1\right)}|\times 100,$$

where $f_{\varepsilon_{r}}$ is the resonance frequency for the solution with $\varepsilon_{r}$, and $f_{empty}$ is the resonance frequency, when the microfluidic channel is empty.

**Figure 9:**
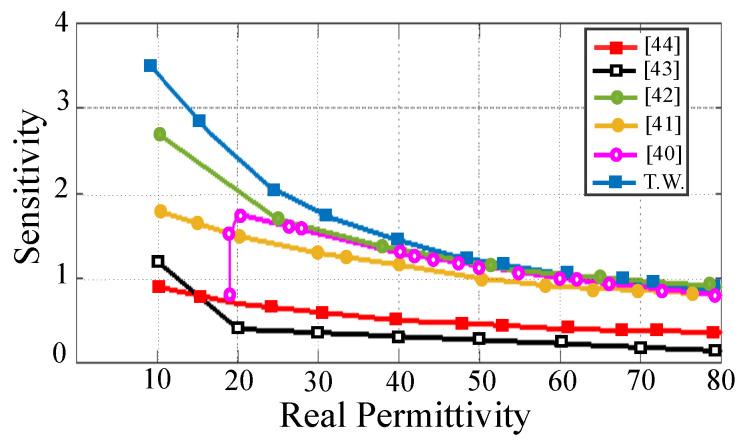
Comparison between the sensitivity of the designed sensor and the state-of-the-art planar devices versus the permittivity of the test samples.

Figure 9 represents the sensitivity of the state-of-the-art sensors as a function of the real permittivity of the test samples. The figure shows that the proposed sensor offers higher sensitivity compared to all of the previously reported designs in the literature. This is mainly due to an optimized design removing any parasitic capacitances contributing to the sensitivity degradation. Table 1 represents a comparison between the proposed sensor and the state-of-the-art designed sensors found in the literature in terms of essential sensing metrics, such as operational frequency, the required amount of sample for testing, the device profile, and the average sensitivity. The liquid-under-test (LUT) is in contact with the device in all of the sensors in the comparison table except Reference [52], where a tiny tube containing the LUT is attached to the sensing area. Based on the table, the proposed sensor offers the highest average sensitivity and the smallest sample for measurement. Although the sensor in Reference [49] has a smaller size than the others, it requires a relatively large amount of sample for testing. The sensors in References [50,51] offer the second and third largest average sensitivities. However, the measured notch depths in these sensors are very small (less than 1.5 dB in Reference [50] and less than 6 dB in Reference [51]). This means that such sensors are not appropriate for characterization of high loss samples. On the other hand, the proposed sensor preserves 10 dB notch depth, even for a very lossy sample, such as ethanol, showing a high capability in measuring samples with highly lossy dielectric properties.

## 4. Conclusions

A very high-sensitivity microwave microfluidic sensor has been designed in this article using a microstrip line loaded with a series connected shunt LC resonator. A very high sensitivity is achieved by eliminating any extra capacitance not contributing to the sensing mechanism. The only capacitive part remaining is the gap capacitance between the triangular patches of the resonator on the top surface of the dielectric substrate. The microfluidic channel is attached along the whole gap area, which preserves the microfluidic samples’ consistent volume and shape during the measurements. A prototype of the sensor has been fabricated and validated with experiments. First, an approximated mathematical model is derived based on water/ethanol mixtures of various concentrations. Then, the mathematical sensing model is verified with water/methanol mixtures of various concentrations. A sensitivity comparison with the state-of-the-art microwave sensor designs shows higher sensitivity of the proposed sensor in comparison with the state-of-the-art sensors in literature, while requiring a very small amount of the sample under the test.

## Figures and Tables

**Table 1:** Comparison between various resonance-based planar microfluidic sensors.

Ref.	Res. Type	f${}_{\mathrm{empty}}$ (GHz)	S.V. (µL)	Rel. Size ($\lambda_{g}^{2}$)	Avg. Sens. (%)
[49]	IDE-SRR	1.06	68	0.11 × 0.053	0.635
[50]	CSRR	2.226	0.52	0.44 × 0.25	0.98
[51]	M-CSRR-CELC	2.45	1.674	0.13 × 0.095	1.44
[52]	SRR	2.6	5	0.23 × 0.08	0.27
[53]	SIR	1.91	0.39	0.1 × 0.09	0.635
[56]	CSRR	1.618	3.9	0.41 × 0.17	0.626
T.W.	Shunt LC	2.7	0.295	0.10 × 0.13	1.61

## Data Availability

Data are contained within the article.
